# First Evidence of Past and Present Interactions between Viruses and the Black Soldier Fly, *Hermetia illucens*

**DOI:** 10.3390/v14061274

**Published:** 2022-06-11

**Authors:** Robert D. Pienaar, Clément Gilbert, Carole Belliardo, Salvador Herrero, Elisabeth A. Herniou

**Affiliations:** 1 Institut de Recherche sur la Biologie de l’Insecte, UMR 7261 CNRS—Université de Tours, 37200 Tours, France; robert.pienaar@univ-tours.fr; 2 Department of Genetics, University Institute of Biotechnology and Biomedicine (BIOTECMED), Universitat de València, 46100 Valencia, Spain; salvador.herrero@uv.es; 3 CNRS, IRD, UMR Evolution, Génomes, Comportement et Ecologie, Université Paris-Saclay, 91198 Gif-sur-Yvette, France; clement.gilbert@egce.cnrs-gif.fr (C.G.); carole.belliardo@univ-cotedazur.fr (C.B.)

**Keywords:** black soldier fly, *Hermetia illucens*, *Totiviridae*, virus discovery, endogenous viral elements

## Abstract

Black soldier flies (BSFs, *Hermetia illucens*) are becoming a prominent research model encouraged by the insect as food and feed and waste bioconversion industries. Insect mass-rearing facilities are at risk from the spread of viruses, but so far, none have been described in BSFs. To fill this knowledge gap, a bioinformatic approach was undertaken to discover viruses specifically associated with BSFs. First, BSF genomes were screened for the presence of endogenous viral elements (EVEs). This led to the discovery and mapping of seven orthologous EVEs integrated into three BSF genomes originating from five viral families. Secondly, a virus discovery pipeline was used to screen BSF transcriptomes. This led to detecting a new exogenous totivirus that we named hermetia illucens totivirus 1 (HiTV1). Phylogenetic analyses showed this virus belongs to a clade of insect-specific totiviruses and is closely related to the largest EVE located on chromosome 1 of the BSF genome. Lastly, this EVE was found to express a small transcript in some BSFs infected by HiTV1. Altogether, this data mining study showed that far from being unscathed from viruses, BSFs bear traces of past interactions with several viral families and of present interactions with the exogenous HiTV1.

## 1. Introduction

Among other entomopathogens, insect viruses appear to have plagued the insect rearing industry for over two centuries [1]. Although well established in Asia, upscaling cricket farming in North America and Europe has been hampered by outbreaks of cricket-infecting viruses belonging to the *Parvoviridae*, *Iflaviridae,* and *Iridoviridae* families that can cause a high level of mortalities and economic losses [2,3,4,5,6]. Besides recent virus discoveries in crickets, there is also evidence of a wide variation in viral prevalence among cricket populations [3,4,5,7]. Similarly, in honey bees, *Apis mellifera*, viral pathogens affect both wild hives and apiaries globally [8]. These examples highlight the potential threats that insect mass-rearing facilities could encounter from insect-infecting viruses. To rapidly circumvent future epizootics, it is important to improve knowledge on the viruses that could emerge in insect mass-rearing facilities, especially in models for which basic information is lacking [1,2].

The black soldier fly (BSF, *Hermetia illucens*, Stratiomyidae) is one such insect species for which mass-rearing is currently undergoing fast worldwide growth [9,10,11] and from which no viruses have so far been described. Most research on BSFs focuses on rearing optimization and application as a prominent source of proteins for the food and feed industry [9,10,12,13], as well as in biotechnology [12,13,14,15,16,17]. BSFs appear particularly robust and resistant to diseases. However, experimental laboratory infections using entomopathogenic nematodes, bacteria, and fungi can cause symptoms and mortality in BSFs [18,19,20,21]. But specific pathogens, including viruses, naturally infectious to BSFs have yet to be characterized [13]. As reports of BSFs mortality are increasing, they demonstrate the need to investigate the virome of BSFs.

As a first effort to characterize the BSF virome, we searched for virus-derived sequences in publicly available genomic and transcriptomic datasets. Such an approach has proven useful when characterizing new, free circulating, exogenous viruses (EXVs), which may be co-sequenced with that of the host [22,23,24,25]. Screening host genomes may also lead to identifying endogenous viral elements (EVEs), i.e., complete or fragmented viral genomes that became integrated into the genome in the germline of their host and were vertically transmitted [23,26]. The characterization of EVEs within a robust paleovirological framework can yield unique insights into historical host-virus interactions [26,27,28,29,30]. Insect genomes can host numerous and diverse EVEs, including some domesticated EVEs that now fulfil key cellular functions [31,32]. Here we took a two-step bioinformatics approach to explore the viruses that BSFs have encountered in the past (EVEs) or that currently infect BSFs (EXV) [23,24,26]. In particular, this study primarily uses in silico analyses of publicly available BSF genomes and transcriptomes to ask: (1) whether there is evidence of viral endogenization in BSF genomes, and (2) whether any of these endogenized viruses could be related to any exogenous viruses found in BSF transcriptomes.

## 2. Materials and Methods

### 2.1. Datasets and Samples

Publicly available black soldier fly transcriptomes were downloaded from the NCBI SRA database (https://trace.ncbi.nlm.nih.gov/Traces/sra/, accessed between 30 December 2020 and 1 October 2021). Samples were from the following bioprojects: PRJEB19091 [33], PRJEB39181 [34], PRJNA431833, PRJNA432297, PRJNA506627 [35], PRJNA573413 [36] and PRJNA575900 [37]. SRA toolkit (v2.10.9, [38]) was used to convert the SRA files and separate their sequence reads into forward and reverse read fastq files. Publicly available BSF genome assembly fasta files GCA_001014895.1 (BGA1) [39], GCA_009835165.1 (BGA2) [36] and GCF_905115235.1 (BGA3) [40] were also retrieved from NCBI assemblies and RefSeq repositories [41]. Of note, BGA3 is assembled at a chromosomal level and includes seven chromosomes and the mitochondrial genome, leading to a total of 1.01 Gb in size and is predicted to be 98.6% complete. The length of the chromosomes themselves ranges from 15.4 to 222.1 Mb, with an N50 value of 180.46 Mb for scaffolds and 16.01 Mb for contigs [40]. Altogether the genome assemblies and transcriptomes represent BSF colonies from widespread origins. They were generated from BSFs reared in China [35,36,37], Germany [33], Italy [34], the United Kingdom [40], and the United States of America [39]. In addition, BGA2 was also generated alongside transcriptomes produced in bioproject PRJNA573413 [36].

### 2.2. Screening BSF Genome for EVEs

Virus-like sequences were screened for in BSF genome assemblies BGA1, BGA2, and BGA3, except for *Retroviridae* and *Hepadnaviridae* using a DIAMOND-python- and R-based pipeline (archived on Zenodo https://doi.org/10.5281/zenodo.6554302, accessed on 16th May 2022). Sequence regions with viral hits according to the NCBI Identical Protein Groups (IPG) database (8 January 2021) were extracted. Endogenous viral element hits from the same family and closer than 50 bp to each other were considered to correspond to a single EVE, and then screened against the full NCBI nr database (22 January 2021) [41]. An R-script (R v4.0.3, [42]) was used to summarize the results and obtain the taxonomical information of each EVE candidate using the packages ‘taxonomizr’ (v0.5.3, [43]) and ‘data.table’ (v1.13.6, [44]). EVE candidates were checked against *Drosophila melanogaster* proteins in the UniProtKB/Swiss-Prot database (30 March 2021) using BLASTx on NCBI for false-positive assessment. Only sequences that did not receive a *D. melanogaster* protein hit were retained as EVE candidates. Afterwards, to determine EVE locations on the BSF chromosomes, they were mapped onto BGA3 using the in-house mapping software of Geneious Prime (v2021.1-2022.02, https://www.geneious.com, accessed on 29 April 2022)). To assess the level of identity between related EVE sequences, if one EVE sequence mapped to a chromosome after the first round of mapping, 20 kb regions which contained the EVE site were extracted from the genome sequence and then the EVEs were remapped to each of the extracted regions. The Geneious mapping parameters were set to the highest sensitivity, but also allowing for any structural variants, short insertions and deletions of any size while excluding any fine-tuning. Finally, to confirm the genomic origin of the EVE sequences, the EVE sequence outputs from the EVE pipeline were mapped to their originating contigs/scaffolds to obtain flanking sequences from the BSF genome of at least 50 bp, depending on contig size. To assess orthology of EVE locations, a megaBLAST on Geneious Prime was then used to determine if the EVE sequences and their flanking regions were found on BGA1, BGA2 and BGA3.

### 2.3. Exogenous Virus Discovery Using Transcriptomic Data

Quality checking of forward and reverse reads was performed using FASTQC (v0.11.9, [45]). Trimmomatic (v0.39, [46]) was then used to filter reads and remove Illumina adapters. Contigs were then assembled using rnaSPAdes (v3.15.2, [47,48]). Virsorter2 (v2.1, [49]) was used to identify and extract viral-like sequences from the SPAdes assembled contigs, followed by using CheckV (v0.7.0, [50]) to assess the estimated completeness and accuracy of the viral-like sequences. CheckV results were searched for sequences that firstly had no warning messages, and for sequences with predicted virus genes. BLASTx (RRID:SCR_001653) was then used on sequences that were considered to be viral by CheckV to screen for false positives and to identify the closest hits against the NCBI ‘Non-redundant protein sequences’ database [41]. The default settings were used: 100 Max target sequences, parameters automatically adjusted for short input sequences, an expected threshold of 0.05, word size of 6, BLOSUM62 matrix, gap costs of existence: 11 and extension: 1 and with a conditional compositional score matrix adjustment and a filter for low complexity regions.

Open reading frames (ORFs) were annotated using Geneious Prime. These ORFs were translated to proteins, and conserved regions were searched using BLASTp (RRID:SCR_001010) with the same parameters as BLASTx, but without filtering for low complexity regions. To identify conserved regions, the E-value threshold was set to 0.01 with a maximum number of hits set to 500 against the CDSEARCH/CDD database.

### 2.4. Phylogeny of Totiviridae

On Geneious Prime, the GAG and POL ORFs of the selected totiviruses were reannotated as per the majority of annotated genomes. Afterwards, the translated amino acid (AA) residues of the POL and GAG ORFs were aligned using the MAFFT aligner (v7.45, [51]) using the G-INS-i algorithm, a BLOSUM62 scoring matrix, and the default values of 0.123 for offset value and 3 for the gap open penalty. The alignments were trimmed at both ends, and alignment columns were retained if at least 10% of the sequences had an amino acid at that position. The alignments were concatenated. A maximum likelihood phylogenetic tree was reconstructed using the IQ-TREE 2 software (v2.1.3, [52]), which allows for the selection of the best-fit evolutionary model for the data using the automated ModelFinder [53] and robustness assessment by ultrafast (UF) bootstrap (1000 iterations) [54] and Shimodaira-Hasegawa-like approximate likelihood ratio test (SH-aLRT) [55]. The tree was visualized using Geneious Prime.

### 2.5. Molecular Validation of EVE

Genetic material from three BSF larvae originating from three rearing facilities was extracted using the ZymoBIOMICS DNA/RNA Miniprep Kit (cat. R2002, ZYMO Research, Freiburg im Breisgau, Germany), and DNA was used for the PCR screening of TotiEVE and to target the region which appears in some transcriptomes. To determine whether the endogenized *Totiviridae* sequence was present in available BSF colonies, the TotiEVE sequences, and hermetia illucens toti-like virus 1 (HiTV1) contigs were mapped to the BSF reference genome (GCF_905115235.1). Primers were designed using Primer 3 (v2.3.7, [56]) on Geneious Prime with settings for Tm between 50 and 58 °C and GC content between 40 to 60 %, and GC clamp to 1 (Table S1). The max size of the target regions searched was 1000 bp, and 650 bp for the region where the short transcripts align (TotiEVE-STs). Amplification reactions were set up with 2.3 µL of 10x Diamond *Taq*^®^ reaction buffer, 1.5 mM of Diamond *Taq*^®^ MgCl_2_ solution, 1 U of Diamond *Taq*^®^ (TAQ-I021, Eurogentec, Liège, Belgium), 0.3 µM per each of forward and reverse primers, 5 µmol of each dNTP (NU-0010, Eurogentec, Liège, Belgium), 7 to 15 ng of DNA template, and filled to a total volume of 25 µL with RNase/DNase Molecular grade water, which was also used as a negative control. Thermocycling was as follows: Initial denaturing of 95 °C (5 min), then 30 cycles of 94 °C (30 sec), 50/52 °C (30 sec), and 72 °C (1 min), followed by a final extension of 72 °C (7 min) (Table S1).

Amplified samples were migrated on an E-Gel^®^ EX 1% Agarose gel alongside a 1 kb Plus Express DNA Ladder (G401001 and 10488091, Invitrogen, Waltham, MA, USA). The bands of the anticipated size were cut out and extracted using the Thermo Scientific^TM^ GeneJET Gel Extraction Kit (K0692, Thermo Fisher Scientific, Waltham, MA, USA). Gel purifications were quantified on a Qubit™ 2.0 Fluorometer with the 1X dsDNA High Sensitivity (HS, Q33231, Invitrogen, Waltham, MA, USA). From the extracted products, 0.41 to 7.76 ng of amplified product DNA underwent a second PCR amplification using the same PCR conditions as mentioned above. Unpurified products from the second PCR amplification were sent to Eurofins Genomics (Konstanz, Germany) for multi-directional sequencing on an ABI 3730XL sequencer (Thermo Fisher Scientific, Waltham, MA, USA). The raw sequence chromatograms were curated on Geneious Prime by aligning them to the reference genome for BSFs, interrogating any mixed peaks, and cross-referencing the bases with the alignments.

## 3. Results and Discussion

### 3.1. Orthologous EVE Sequences Found in Three BSF Genomes

Screening the three BSF genome assemblies using our bioinformatic pipeline revealed 27 viral sequences with close BLASTx hits to members of six viral families (Table 1, Tables S2 and S3). The size of the viral sequence hits ranged between 148 and 3750 nucleotides. All three BSF genome assemblies received hits related to *Partitiviridae* (5), *Parvoviridae* (7), *Totiviridae* (10), and *Totiviridae*-like (3) viruses, while a hit related to either *Rhabdoviridae* or *Xinmoviridae* was found in BGA2 and BGA3 respectively. The pipeline also identified six sequences of porcine reproductive and respiratory syndrome virus (*Arteriviridae*) in the data from BGA2, but these were discarded because they were on contigs that all lacked flanking insect sequences and therefore likely resulted from sequence contamination. 

**Figure 1:**
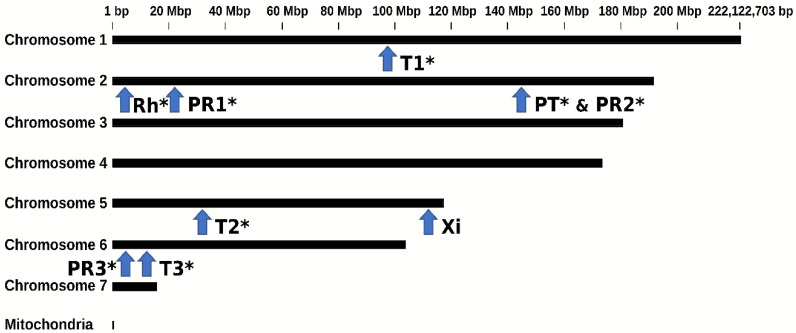
Positions of EVE candidates in the genome of BSF, assembled at the chromosome level (BGA3). Letters represent specific sites of related EVE sequences. The letters represent sites where certain EVE sequences obtained from BGA1, 2 & 3 that were related to *Partitiviridae* (PT), *Parvoviridae* (PR1, PR2, PR3), *Rhabdoviridae* (Rh), *Totiviridae* (T1, T2, T3) and *Xinmoviridae* (Xi) could be mapped. The asterisks indicate which EVE site was found on at least two of the three BGAs. Sequences can be found in Table S3.

The viral hit retrieved by the pipeline corresponded mostly to *capsid* (17) and *RNA-dependent RNA polymerase* (*RdRP*) (8) genes (Table 1 and Table S2). There was a high nucleotide identity between the EVEs of the same viral families found in the three genomes (between 50 and 100%; Table S2). This suggested that some EVEs may be orthologous and predate the separation of the BSF populations from which the genomes originated. As BGA3 was assembled to chromosome level [40], it was possible to infer the precise location of each of the nine EVEs on the BSF genome (Figure 1, Table S2). Overall, the EVEs were distributed on four of the seven BSF chromosomes (Figure 1; Table 1 and Table S2).

Mapping showed that EVE sequences with above 98% identity to TotiEVE T1 were flanked by the same BSF genome sequences (Table S2). Furthermore, successful PCR amplifications were obtained from DNA of larvae coming from three independent BSF colonies using primers to target regions within TotiEVE T1, outside of TotiEVE T1, and overlapping TotiEVE T1 and its flanking region (Figure 1). Between data mining analyses and molecular analyses, this totivirus EVE TotiEVE T1 was found in BSF strains reared in the United States of America, United Kingdom, France, and China. This strongly suggests that TotiEVE T1 is present and orthologous in several reared BSF populations.

Likewise, EVE orthology in the three BSF genomes was confirmed through megaBLAST analyses of EVE sequence contigs with their 5′/3′ flanks, as well as the nine BGA3 20 kb BSF regions, including each EVE. TotiEVE T1, T2 and T3, PartitiEVE PT, and RhabdoEVE Rh were orthologous in the three BSF genomes (Figure 1). ParvoEVE PR 1 and PR3 were shared at orthologous positions in BGA 2 and 3, while ParvoEVE PR2 showed orthology for BGA 1 and 3. XimnoEVE Xi was only found in BGA3 (Table 1 and Table S2). An additional six virus-like sequences (PartitiEVE2G1, TotiEVE1G1, TotiEVE2G1, TotiEVE10G2, ParvoEVE3G2, PartitiEVE3G2) were found in BGA1 and 2, but could not be located on BGA3, although they were 50 to 75% identical to some of the BGA3 EVEs. They could either be specific to the genomes they were found in or have been removed from the final BGA3 assembly.

This paleovirological analysis shows that the ancestor of the BSF populations from which the data originated (USA, China, UK) already harboured at least nine EVEs from the *Totiviridae, Parvoviridae, Partitiviridae, Rhabdoviridae,* and *Xinmoviridae* families. These are genomic traces of past infections showing that BSFs have had interactions with exogenous viruses from these families. As three EVE loci each were detected for parvoviruses and totiviruses, and since the relatively low similarities between them (up to 75%) suggest independent endogenization, interactions between BSFs and exogenous viruses from *Parvoviridae* and *Totiviridae* may have been recurrent over evolutionary time (Figure 1, Table 1 and Table S2).

### 3.2. Description and Phylogeny of HiTV1, an Exogenous Totivirus

As with 3750 nucleotides in length, TotiEVE T1 was the largest EVE we found in the BSF genome and comprised partial sequences of both capsid (GAG) and RDRP (POL) genes. We hypothesized that BSF interactions with totiviruses may have happened more recently than with other viruses. We thus investigated the presence of TotiEVE T1 relatives in BSF transcriptomes. The exogenous virus discovery pipeline provided a list of virus candidate contigs, which had leptopilina boulardi toti-like virus (LbTV) as the closest related hit, and this was validated by reciprocal BLASTx. These contigs were labelled as sequences of a new virus candidate, hermetia illucens toti-like virus 1 (HiTV1).

**Figure 2:**
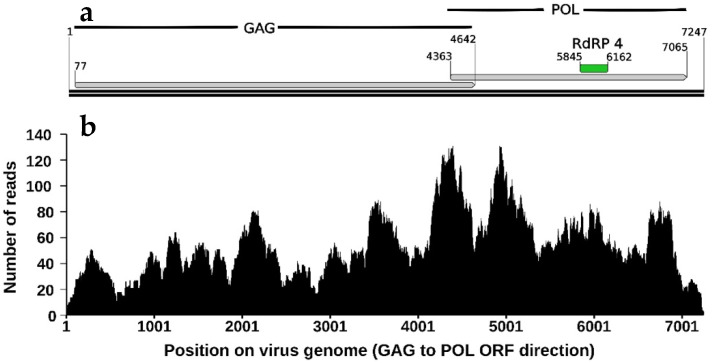
Annotation of HiTV1 genome sequence (**a**) and read coverage (**b**). (**a**) Numbers represent the nucleotide position, and two long horizontal black lines represent the double-stranded RNA se-quence. A conserved RdRP 4-like domain (E-value of 1.46 × 10^−4^, PF02123) was annotated in green. (**b**) Using Bowtie2 [60] and SAMtools [61], raw sequence reads from the transcriptome SRR14339788 were mapped onto the HiTV1 contig 1, which resulted from a SPAdes assembly of SRR14339788 reads. The average coverage of the reads across the contig without gaps was 53.79x.

**Figure 3:**
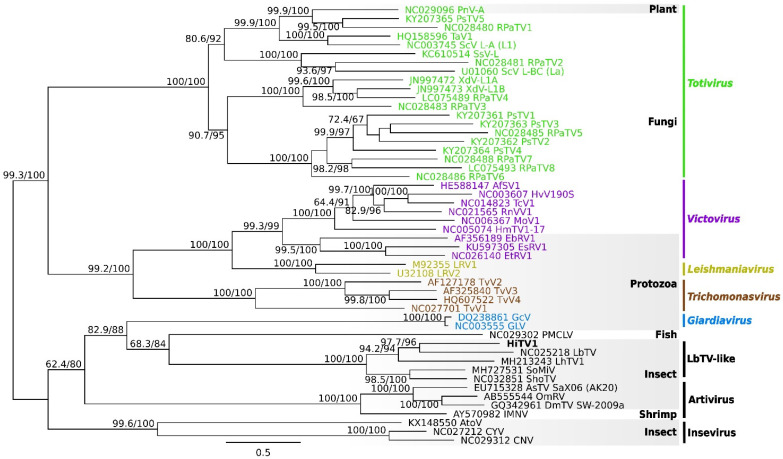
Phylogenetic relationships of HiTV1 within the family *Totiviridae*. The maximum likelihood tree is based on the concatenated alignment of POL and GAG sequences. Phylogenetic robustness was assessed using Shimodaira Hasegawa-like approximate likelihood ratio test and UF bootstrap; values are reported for each node. Host groups and virus genera are reported to the right side of the tree. Hermetia illucens toti-like virus 1 (HiTV1) is highlighted in bold in the tree. Virus clades belonging to genera, currently recognised by the International Committee on Taxonomy of Viruses (ICTV) are coloured for the illustrative purpose of highlighting that HiTV1 belongs to a new totivirus genus. Full names of abbreviations can be found in Table S4.

In the transcriptome SRR14339788, derived from larval gut (Table 2), a HiTV1 contig 1 of 7247 nt in length was found and subsequently used as the reference sequence for annotation (Figure 2) and phylogeny (Figure 3). *Totiviridae* genomes are double-stranded RNA and normally between 4.6 to 7 kbp in length [57], although genomes of *Totiviridae* such as LbTV (NC_025218.2) and papaya meleira virus (NC_028378.1), can be as long as 8021 bp and 8768 bp, respectively. Like most *Totiviridae* [57], HiTV1 has a simple genome consisting of a GAG and a POL ORF, which encode for a capsid protein and an RdRP protein (Figure 2). The POL ORF contains an RdRP 4-like domain (PF02123), typically associated with viral families such as *Totiviridae* [58,59]. Read mapping of transcriptome SRR14339788 onto the HiTV1 contig 1 showed an average coverage of 53.79x across the length of the viral genome (Figure 2), which gives high confidence in the HiTV1 assembly.

Out of the 65 BSF RNAseq datasets screened, HiTV1 was found as nearly complete genome contigs in five transcriptomes from three distinct bioprojects, and as shorter contigs in another 48 transcriptomes (Table S3f). This finding suggests that HiTV1 is not a sequence contaminant but rather a genuine virus that may be found in particular BSF colonies. As these transcriptomes originated from BSFs reared in Germany, Italy, and China, HiTV1 appears globally distributed. (Table 2 and Table S3a). Moreover, HiTV1 was found in the gut of individual BSF larvae reared under different conditions, a pool of five larvae, and even BSF eggs and adult antennae (SRR10233312.1).

The amount of HiTV1 RNA, which corresponds to the viral genome titre plus viral genome expression, was evaluated by mapping RNAseq reads. TPM values for gag and pol were quite similar in each sample, showing that both HiTV1 genes were present at similar levels, although more often slightly higher for pol (Table 3). Comparing these values to that of the Actin-5C gene, which is constitutively expressed in BSFs, showed that in all larval samples (contigs 1 to 4), HiTV1 only reached a ratio of 0.002–0.009 of RNA abundance, whereas in the egg mass (contig 5) this ratio reached 0.369–0.434 (Table 3). Several hypotheses could be invoked to explain the different read counts and the ratio between larval and egg stages: (1) viral titre might be higher in the BSF colony the egg mass came from; (2) viral infection might be restricted in particular cells and thus viral reads diluted in larval BSF transcriptomes; (3) generally, the level of actin expression might be lower in the eggs compared to fully active larvae, and this could inflate the ratio (4) HiTV1 particles might accumulate in the eggs. Further work would be needed to determine the tissue tropism of HiTV1, although it was already found in dissected larval midguts, adult antennae, and egg masses.

Phylogenetic analyses based on the concatenated alignment of the GAG and POL ORFs show that HiTV1 belongs to a clade comprising previously discovered insect-associated totiviruses (Figure 3), including LbTV1 (from *Leptopilina boulardi*, wasp), LhTV1 (from *Linepithema humile*, ant), SoMIV (from *Solenopsis invicta*, ant) and ShoTV (from a pool of insects) [23,63,64,65]. This clade is highly supported and may constitute a new totivirus genus. It is related to PMCLV, which is found in fish, and to the *Giardiavirus* genus, within a larger group of totiviruses predominantly associated with arthropods (Figure 3). This group falls outside the clade formed by the genera *Totivirus*, *Trichomonasvirus*, *Victorivirus,* and *Leishmaniavirus*—which mainly infect protozoa or fungi [57]. The host range of *Totiviridae* was historically thought to be restricted to fungi and protozoa but is continuously expanding with the discovery of novel *Totiviridae*-like sequences in Arthropoda [57,66]. This is also pushed by the growing association with the prevalence of *Totiviridae*-like contigs appearing in arthropod (and bat faeces) transcriptomes sequenced from samples collected from multiple sites and conditions [23,67,68,69,70], as experienced in this study. Furthermore, experimental studies have demonstrated that arthropod hosts, either in cell culture or whole models, can be infected by totiviruses [63,67,68,70,71]. Altogether, the phylogenetic analyses support the fact that HiTV1 is a totivirus that belongs to a clade that infects insects, and therefore it is highly likely that it infects BSF.

### 3.3. Expression of the Endogenous TotiEVE T1

**Figure 4:**
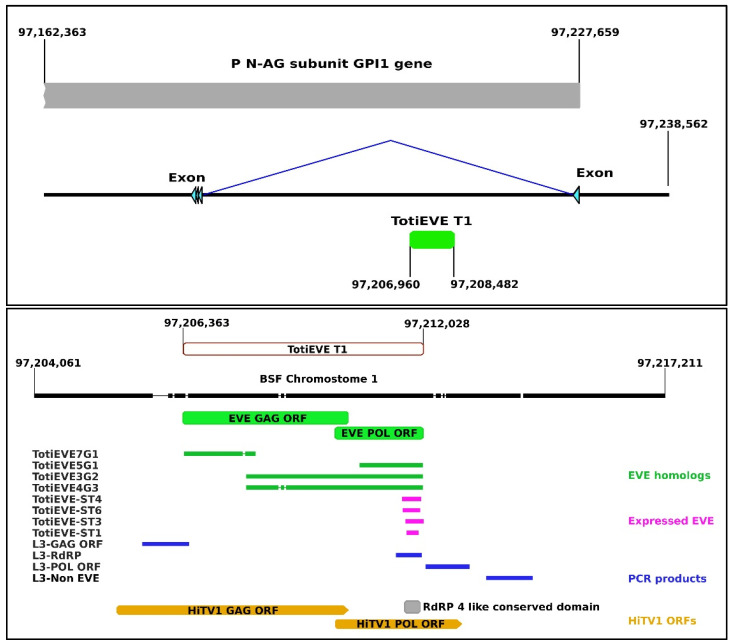
Mapping of Totiviridae-related sequences to TotiEVE T1 on chromosome 1 (black) on the BSF genome. TotiEVE T1 (green) is located between two exon sequences (light blue) of the BSF *Phosphatidylinositol N-acetylglucosaminyltransferase subunit GPI1* gene (P N-AG subunit GPI1 gene) (XM_038053365.1) (grey) (**top**). In the (**bottom**), a closeup of TotiEVE T1 (brown) displaying the mapping of the orthologs found in BGA1, 2, and 3 (green), the expressed short transcripts (pink), the amplified PCR products (dark blue), and an overlay of the HiTV1 ORFS showing that the EVE is shorter than the exogenous virus (orange) and that the expressed EVE aligns to the RdRP conserved domain (grey). The sequences were mapped to a 20 001 nt sequence that flanks TotiEVE T1. The sequences can be found in; Table S3.

**Figure 5:**
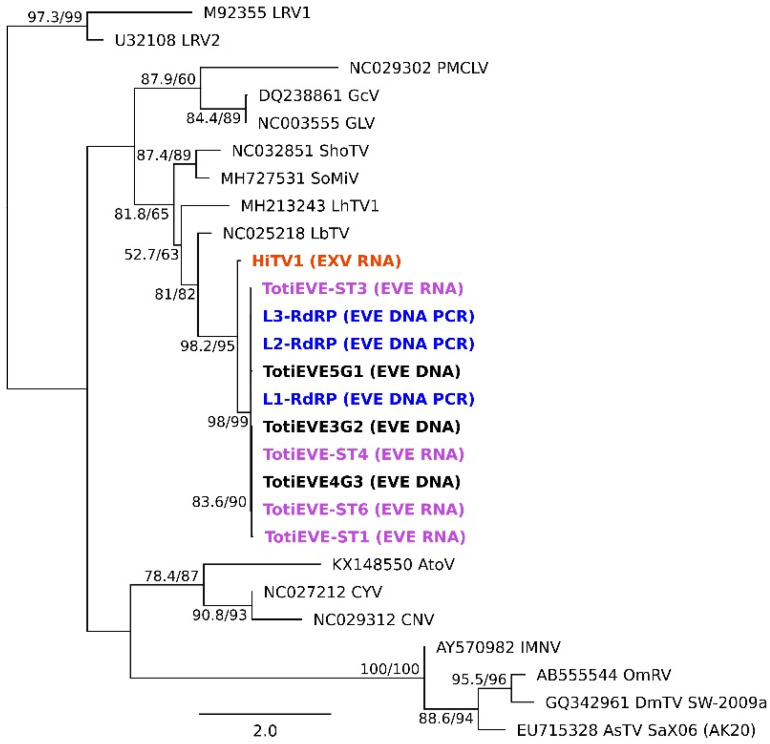
Phylogeny of HiTV1 and endogenous Totivirus sequences located in T1 on BSF genomes. Sequences in bold are associated with this study. HiTV1 is in orange, TotiEVEs located in T1 in the three genomes in black, the expressed TotiEVE ST found in four transcriptomes in purple, and PCR products of T1 from independent BSF samples. The outgroup consisted of LRV1 and LRV2 of the genus *Leishmaniavirus* within the *Totiviridae* family. Branch support for the maximum likelihood tree was in the order of SH-aLRT and UF bootstraps. Node values scoring lower than 50 were not displayed.

Once they have been integrated into the genome of a host, EVE sequences usually remain as degraded fossil traces of past infections. However, some EVEs may become domesticated or exapted, in which case they can be expressed by the hosts [26,31,32,72,73,74]. Short transcripts, termed TotiEVE-ST were found in some transcriptomes (Table S3a,b). Mapping these 243 to 395 nucleotide-long transcripts on the BGA3 genome showed that they derived from the RdRP region of the TotiEVE in locus T1 (Figure 4). These TotiEVE-ST sequences, except TotiEVE-ST2 and 5, completely overlapped (Figure 4). In mosquitoes, EVEs have been found to regulate viral infections through the piRNA system [32]. In BSFs, the TotiEVE-ST was only expressed in transcriptomes where HiTV1 contigs were also found, including as contigs shorter than 5 kb (such as in transcriptomes SRR10158821, SRR14339789, SRR14339790, SRR14339791, SRR14339793, SRR14339795). However, these short transcripts only had an average identity of 71.15% to HiTV1 (Figure 5). Therefore, in the absence of experimental evidence, it is unclear whether they may exert any specific anti-viral activity against this totivirus.

### 3.4. Phylogenetic Relationships between the Exogenous HiTV1 and TotiEVEs

To investigate the genetic diversity found in the TotiEVE of locus T1 in relation to HiTV1, all the sequences detected in the genomes and transcriptomes through our bioinformatics pipeline or by PCR were included in a single alignment to determine their interrelationships regarding other totiviruses (Figure 4). The phylogenetic analysis revealed that HiTV1 was the sister group of all the EVE sequences, which were more than 97% identical to one another, apart from TotiEVE-ST4, which was 92% identical to the others (Figure 4 and Figure 5). This result suggests that HiTV1 is closely related to the exogenous ancestor of the orthologous TotiEVEs located at the locus T1 on chromosome 1 of genomes in all the BSF populations investigated.

## 4. Conclusions

As the capacity for rearing black soldier flies develops worldwide, epidemiological models predict it is likely that pathogen outbreaks, including viruses, will occur [75]. No viruses have so far been discovered in BSFs. However, there is already a wealth of genomic and transcriptomic data that has been generated by different studies and is publicly available. Using public data as a starting point for metagenomic and metatranscriptomic approaches for discovering viruses has led to the discovery of a large wealth of RNA viruses [23,24,76], including in insects [22,25,77]. One major point arising from these discoveries has been the difficulty in determining if viruses can infect the organism in which they were discovered in the absence of small RNAseq data or laboratory experiments [24,25,78]. Exploring EVEs present in the BSF genome offered additional insights into the virome of BSFs [26,27,79]. The EVE results showed that members of five different viral families which are known to infect insects [63,80,81,82,83,84,85] have interacted with BSFs in the past. The clustering of HiTV1 among arthropod infecting *Totiviridae* and its presence across BSFs under different rearing conditions and locations provides strong evidence that BSFs are the natural hosts of this virus. Remarkably, the TotiEVE was found to produce a short transcript. The function of this TotiEVE-ST remains unclear. However, its presence alongside infections of HiTV1 could indicate that it might be involved in the immune response of BSFs against HiTV1. In conclusion, this study presents the first evidence of past and present virus interactions with BSFs.

## Figures and Tables

**Table 1:** Summary of EVE sequences found in three BSF genomes.

Viral Family	EVE		BGA	Best Viral Hit	Viral Hit Similarity		Coordinates on BGA Contigs ^§^
	Name	Location ^†^			AA (%) ^¤^	Protein ^#^	
*Partitiviridae*	PartitiEVE	PT	1	Atrato Partiti-like virus 2	44.6	Capsid	JXPW01014295.1:343-1512
		np*	1		49	Capsid	JXPW01121853.1:492-1707
		np*	2		47.5	Capsid	VFFH01000694.1:2871386-2872788
		PT	2		54.8	Capsid	VFFH01002716.1:5535-6390
		PT	3		54.1	Capsid	LR899010.1:144460311-144461333
*Parvoviridae*	ParvoEVE	PR1	2	Clinch densovirus 1	66.7	Capsid	VFFH01002420.1:17403-17642
		PR1	3	*Densovirinae* sp.	39.2	Capsid	LR899010.1:21909312-21909550
		PR2	1	Haematobia irritans densovirus	45.3	Capsid	JXPW01295709.1:732-1063
		np*	2		62.5	ORF1	VFFH01002716.1:27731-27993
		np*	2		33.5	Capsid	VFFH01002716.1:22618-23330
		PR2	3		45.4	Capsid	LR899010.1:144484849-144485180
		PR3	3	Lone star tick densovirus 1	45.8	ORF1	LR899014.1:3976151-3976299
*Rhabdoviridae*	RhabdoEVE	Rh	2	Entomophthora rhabdovirus A	55.1	RdRP	VFFH01000694.1:2885224-2885413
*Totiviridae*	TotiEVE	T1	1	Leptopilina boulardi toti-like virus	54.8	RdRP	JXPW01175605.1:2029-3362
		T2	1		34.6	Capsid	JXPW01052892.1:5735-9302
		T1	1		28.6	Capsid	JXPW01318472.1:69-1591
		T3	1		33.2	Capsid	JXPW01168285.1:326-1578
		T1	2		53	RdRP	VFFH01002277.1:524489-528239
		T2	2		30.4	Capsid	VFFH01001437.1:1443067-1446431
		np*	2		38.5	Capsid	VFFH01001390.1:32171-33459
		T3	2		36.7	Capsid	VFFH01001777.1:322470-323680
		T2	3		30.4	Capsid	LR899013.1:31694664-31698028
		T3	3		36.8	Capsid	LR899014.1:10621516-10622714
		np*	1	Linepithema humile toti-like virus 1	38.9	Capsid	JXPW01318876.1:130-1861
		T1	3	Dumyat virus	35.4	RdRP	LR899009.1:97208286-97212028
		np*	1		39.9	RdRP	JXPW01237450.1:1885-3346
*Xinmoviridae*	XinmoEVE	Xi	3	Lepidopteran anphe-related virus OKIAV50	61.6	RdRP	LR899013.1:111581535-111582826

^†^ Location refers to the mapping location on the BGA3 genome with the names PartitiEVE (PT), ParvoEVE (PR1-3), TotiEVE (T1-3), RhabdoEVE (Rh) and XimnoEVE (Xi), as illustrated in Figure 1. ^§^ Coordinates on the contigs on the BSF genome assembly (BGA) from which each EVE originated. ^#^ The protein hit was named according to the type of protein that the original hit was associated with. A more comprehensive set of information and sequences can be found in Tables S2 and S3d, respectively. ^¤^ AA stands for amino acid similarity. np* (no position) indicates viral-like sequences related to other EVEs, but that could not be located on BGA3.

**Table 2:** RNAseq datasets containing HiTV1 contigs longer than 5 kb.

HiTV1 Contig	SRA Number (Bioproject)	Sample	Reference
contig 1	SRR14339788 (PRJNA573413)	Midgut of four-day-old larvae reared on food waste	[36]
contig 2	SRR10158821.1 (PRJNA573413)	Midgut of four-day-old larvae reared on cow manure	[36]
contig 3	SRR14339795 (PRJNA573413)	Midgut of eight-day-old larvae reared on cow manure	[36]
contig 4	ERR1801992.1 (PRJEB19091)	Five individual larvae	[33]
contig 5	SRR8242288 (PRJNA506627)	Egg Mass	[35]

**Table 3:** Sequence abundance of HiTV1 compared to BSF Actin-5C in different transcriptomes.

Name	Transcripts Per Million (TPM) ^§^				
	HiTV1 Contig 1	HiTV1 Contig 2	HiTV1 Contig 3	HiTV1 Contig 4	HiTV1 ^†^ Contig 5
HiTV1 *pol*	9343	6192	3416	3590	204,488
HiTV1 *gag*	6848	3473	2302	2494	240,733
*Actin-5C*	983,809	990,335	994,282	993,916	554,779
Ratio *pol*/*Actin-5C*	0.009	0.006	0.003	0.004	0.369
Ratio *gag*/ *Actin-5C*	0.007	0.004	0.002	0.003	0.434

^§^ Transcriptomic reads mapped to each gene and virus contig using HISAT2 [62] were filtered using SAMtools, and the TPM values were calculated for CDS regions using Geneious Prime. ^†^ Contig 5 was found in the transcriptome of an egg mass, suggesting low cellular activity based on the number of reads found for *Actin-5C*.

## Data Availability

Data in this study was mainly generated from publicly available NCBI bioprojects (https://www.ncbi.nlm.nih.gov/bioproject/, accessed on 16 April 2022). The sequence for HiTV1 contig 1 found in Table S3a, is also available in the Third Party Annotation Section of the DDBJ/ENA/GenBank databases under the accession number TPA: BK061373.

## Supplementary Materials

The following supporting information can be downloaded at: https://www.mdpi.com/article/10.3390/v14061274/s1, Table S1: Details of primers used to amplify regions of the TotiEVE T1 (HITE) on chromosome 1 of BSFs; Table S2: Description of BSF EVE candidate sequences found in BSF genomes using the EVE pipeline. and their relation to EVE sites on the BSF genome; Table S3 List of sequences resulting from study and related *transcriptome list.;* Table S4: Abbreviations of virus names or accepted virus species names of those used in phylogenetic trees and paper.; Figure S1: Short fragment detection of TotiEVE sequence and expressed TotiEVE-ST region by PCR amplification of BSF larvae extracted DNA (L) from three different rearing facilities.
