# Supplementary material for: First Evidence of Past and Present Interactions between Viruses and the Black Soldier Fly, Hermetia illucens
Source: Viruses. 2022 Jun 11;14(6):1274. doi: 10.3390/v14061274 (PMC9231314; doi:10.3390/v14061274)
Supplement: Supplementary file 1 [file viruses-14-01274-s001.zip › viruses-1729774-supplementary.pdf]

Table S1 Details of primers used to amplify regions of the TotiEVE T1 (HITE) on chromosome 1 of BSFs.

Primer	Sequence	Region on BSF genome (bp)	Target on TotiEVE	Expected PCR product size (bp)	Annealing temperature (°C)
HITEHNE F & R	5′-GTT GTA ATC GGG AAT TTG GC-3′ & 5′-GAA TGT GAA TAG AAA CTC CGC-3′	97,213,374 – 97,214,373	Host sequence outside EVE	~1000	52
HITEP F & R	5′-AAC AAA ATG TCG CAA CAA GG-3′ & 5′-TAC AGA ATA TCC CAG AGT GC-3′	97,212,055 – 97,213,054	POL ORF end of TotiEVE	~1000	50
HITE F & R	5′-TTA TAA AGG TAC CGC TGG AC-3′ & 5′-TGC TTA TCT ATT GGG CTA GC-3′	97,211,454 – 97,212,021	RdRP-like domain	~568	50
HITEG F & R	5′-ACT CAT GAT CGA AAA GGA CTC-3′ & 5′-TTT CCA AAA CAT CGA TCT GC-3′	97,206,252 – 97,207,251	GAG ORF end of TotiEVE	~1000	50
HITEHNE F & HITEP R	5′-GTT GTA ATC GGG AAT TTG GC-3′ & 5′-TAC AGA ATA TCC CAG AGT GC-3′	97,212,055 – 97,214,373	Host sequence outside EVE to POL ORF	~2300*	52
HITEHNE F & HITE R	5′-GTT GTA ATC GGG AAT TTG GC-3′ & 5′-TGC TTA TCT ATT GGG CTA GC-3′	97,211,454 – 97,214,373	Host sequence outside EVE to RdRP-like domain	~2900*	52

*extension time was changed from 1 minute to 3 minutes.

Table S4 Abbreviations of virus names or accepted virus species names of those used in phylogenetic trees and paper.

NCBI Accession	Virus species/name	Paper abbreviation*
AB555544	Omono River virus	OmRV
GQ342961	Drosophila melanogaster totivirus SW-2009a	DmTV SW-2009a
EU715328	Armigeres subalbatus virus SaX06-AK20	AsTV SaX06 (AK20)
AY570982	Penaeid shrimp infectious myonecrosis virus	IMNV
BK061373	Hermetia illucens Toti-like virus 1	HiTV1
NC025218	Leptopilina boulardi Toti-like virus	LbTV
MH213243	Linepithema humile toti-like virus 1	LhTV1
MH727531	Solenopsis midden virus	SoMiV
NC032851	Shuangao toti-like virus	ShoTV
HQ158596	Tuber aestivum virus 1	TaV1
NC003745	Saccharomyces cerevisiae virus L-A L1	ScV L-A (L1)
KY207365	Puccinia striiformis totivirus 5	PsTV5
NC028480	Red clover powdery mildew-associated totivirus 1	RPaTV1
NC029096	Panax notoginseng virus A	PnV-A
KC610514	Scheffersomyces segobiensis virus L	SsV-L
NC028481	Red clover powdery mildew-associated totivirus 2	RPaTV2
U01060	Saccharomyces cerevisiae virus L-BC La	ScV L-BC (La)
JN997472	Xanthophyllomyces dendrorhous virus L1A	XdV-L1A
JN997473	Xanthophyllomyces dendrorhous virus L1B	XdV-L1B
LC075489	Red clover powdery mildew-associated totivirus 4	RPaTV4
NC028483	Red clover powdery mildew-associated totivirus 3	RPaTV3

KY207361	Puccinia striiformis totivirus 1	PsTV1
KY207363	Puccinia striiformis totivirus 3	PsTV3
KY207362	Puccinia striiformis totivirus 2	PsTV2
KY207364	Puccinia striiformis totivirus 4	PsTV4
NC028485	Red clover powdery mildew-associated totivirus 5	RPaTV5
NC028486	Red clover powdery mildew-associated totivirus 6	RPaTV6
NC028488	Red clover powdery mildew-associated totivirus 7	RPaTV7
LC075493	Red clover powdery mildew-associated totivirus 8	RPaTV8
KX148550	Anopheles totivirus	AtoV
NC027212	Camponotus yamaokai virus	CYV
NC029312	Camponotus nipponicus virus	CNV
NC029302	Piscine myocarditis-like virus	PMCLV
DQ238861	Giardia canis virus	GcV
NC003555	Giardia lamblia virus	GLV
AF356189	Eimeria brunetti RNA virus 1	EbRV1
KU597305	Eimeria stiedai RNA virus 1	EsRV1
NC026140	Eimeria tenella RNA virus 1	EtRV1
HE588147	Aspergillus foetidus slow virus 1	AfSV1
NC003607	Helminthosporium victoriae virus 190S	HvV190S
NC014823	Tolypocladium cylindrosporum virus 1	TcV1
NC021565	Rosellinia necatrix victorivirus 1	RnVV1
NC005074	Helicobasidium mompa totivirus 1-17	HmTV1-17
NC006367	Magnaporthe oryzae virus 1	MoV1
M92355	Leishmania RNA virus 1	LRV1
U32108	Leishmania RNA virus 2	LRV2
AF127178	Trichomonas vaginalis virus 2	TvV2
AF325840	Trichomonas vaginalis virus 3	TvV3
HQ607522	Trichomonas vaginalis virus 4	TvV4
NC027701	Trichomonas vaginalis virus 1	TvV1

*Abbreviations used were for convenience as some viruses do not have official abbreviations.

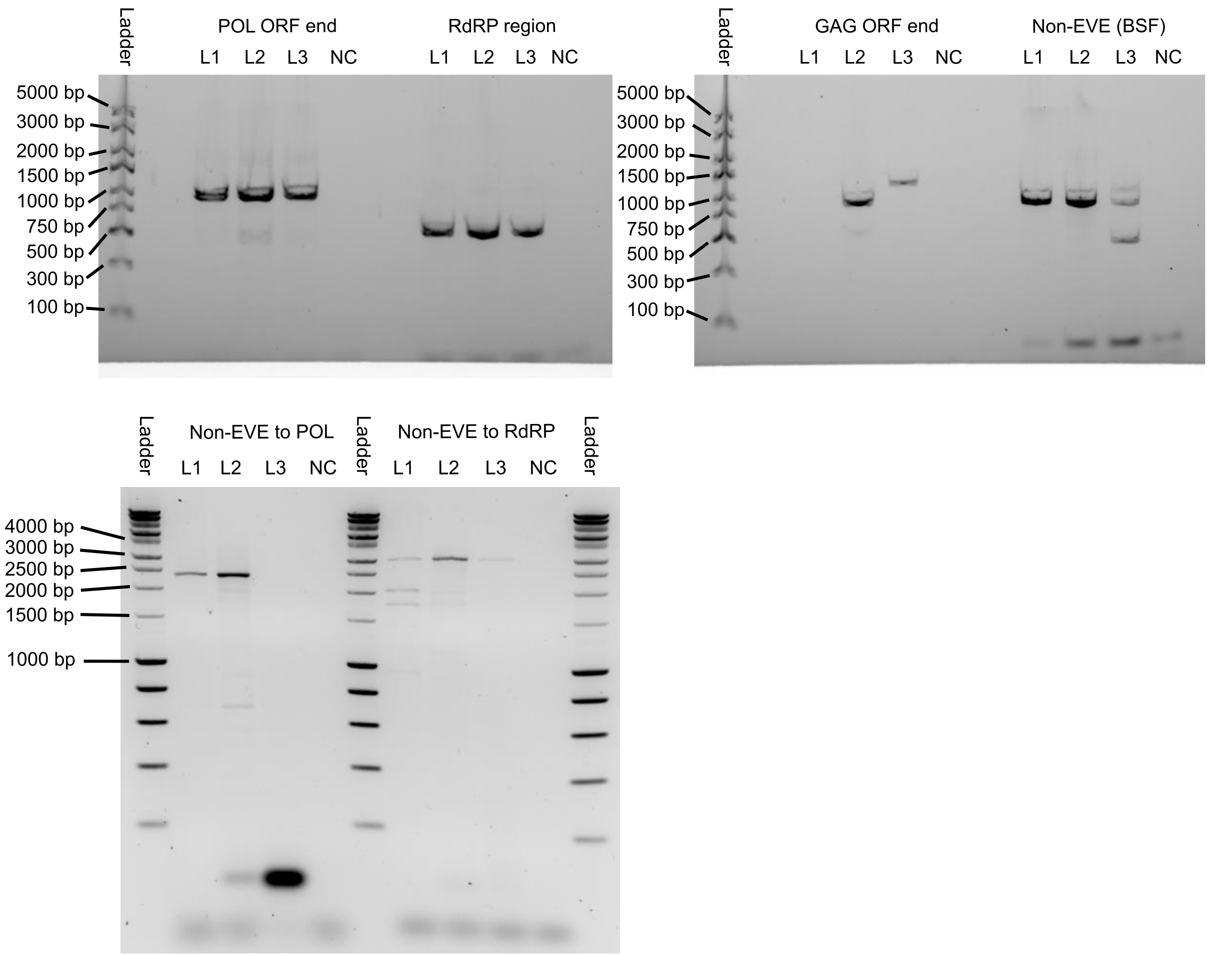

Figure S1 PCR validation of the integration of TotiEVE and expressed TotiEVE-ST within the genome of BSF larvae (L1, L2, L3) originating from three independant rearing facilities. (Top) Primers designed amplified short fragments (~1 kb), firstly at either extremity of the TotiEVE integration site on chromosome 1 according to the 5′ to 3′ orientation of the HiTV1 polymerase (POL) and capsid encoding (GAG) ORFs. Secondly a shorter fragment (~0.5 kb) to cover the region of the RdRP conserved domain-like sequence appearing in BSF transcriptomes and a region of BSF non-viral related sequence, which was outside of the alignment spanned by the HiTV1 contig, near the GAG ORF-like region of the TotiEVE integration. (Bottom) Primer HITEHNE F was used with HITEP R and HITE R to amplify fragments which overlapped the non-EVE BSF flanking sequence with the TotiEVE POL and RdRP ORFs. These fragements were migrated on a 1.2% agarose gel and were sized according to the SmartLadder (MW-1700-10, Eurogentec, Liège, Belgium). Positive bands on the gel demonstrate that the TotiEVE fragments in Larval L1 to L3 are indeed integrated in the BSF genome.

Table S2 Description of BSF EVE candidate sequences found in BSF genomes using the EVE pipeline and their relation to EVE sites on the BSF genome

Inferred location of EVE to BSF chromosome						EVE sequence details								DIAMOND values			Viral hit Classification of EVE sequence						Non-viral hit Classification of EVE sequence			
**Name of Site on Chromosome**	**Pairwise Identity (%)**	**Identical sites (%)**	**Identical sites (nt)**	**BSF chromosome sequence mapped to**	**Coordinates on chromosome**	**BSF genome assembly in paper (BGA)**	**BSF genome assembly accession**	**EVE number**	**Sequence direction**	**BSF contig**	**Co-ordinates on contig (nt)**	**EVE length (aa)**	**EVE length (nt)**	**Viral hit similarity (%)**	**Non-viral hit similarity (%)**	**E-value of virus hit**	**Closest Viral family**	**Viral hit name**	**Confirmed/predicted viral protein**	**Translated ORF protein type hit**	**Co-ordinates on closest virus protein hit (aa)***	**Best viral hit accession**	**Closest non-Viral family**	**Species of non-viral hit**	**Confirmed/predicted non-viral protein**	**Best non-viral hit accession**
T1	98.5	98.5	1314	1	97,210,694-97,212,027	1	GCA_001014895.1	TotiEVE5G1	Reversed	JXPW01175605.1	2029-3362	445	1333	54.8	98.1	4.2×10^{-159}	*Totiviridae*	Leptopilina boulardi Toti-like virus	RdRP	RdRP	68-512	YP_009072448.1	Stratiomyidae	*Hermetia illucens*	Unnamed protein product	CAD7078331.1
PT	87.4	87.4	1023	2	144,460,311-144,461,333	1	GCA_001014895.1	PartitiEVE1G1	Forward	JXPW01014295.1	343-1512	258	1169	44.6	29.9	5×10^{-56}	*Partitiviridae*	Atrato Partiti-like virus 2	Capsid	Capsid	131-339	QHA33903.1	Fabaceae	*Trifolium pratense*	Hypothetical protein L195_g036511 partial	PNX80509.1
np§	53.4	53.4	652	2	144,460,110-144,461,323	1	GCA_001014895.1	PartitiEVE2G1	Forward	JXPW01121853.1	492-1707	255	1215	49	28.8	4.2×10^{-80}	*Partitiviridae*	Atrato Partiti-like virus 2	Capsid	Capsid	101-355	QHA33903.1	Halictidae	*Nomia melanderi*	Uncharacterized protein LOC116434784 isoform X2	XP_031849418.1
np§	66	66	966	1	97,208,027-97,209,308	1	GCA_001014895.1	TotiEVE1G1	Forward	JXPW01237450.1	1885-3346	258	1461	39.9	87.2	1.6×10^{-74}	Unclassified (*Totiviridae* -like)	Dumyat virus	RdRP	RdRP	15-345 (970-1301 LbTV)	QAY29251.1	Stratiomyidae	*Hermetia illucens*	Unnamed protein product	CAD7078329.1
T1	99.1	99.1	1510	1	97,206,960-97,208,482	1	GCA_001014895.1	TotiEVE7G1	Forward	JXPW01318472.1	69-1591	471	1522	28.6	73.2	4.3×10^{-36}	*Totiviridae*	Leptopilina boulardi Toti-like virus	Capsid	Capsid	870-1004	YP_009072447.2	Stratiomyidae	*Hermetia illucens*	Unnamed protein product	CAD7090177.1
T2	89.2	89.1	3186	5	31,694,663-31,697,875	1	GCA_001014895.1	TotiEVE6G1	Reversed	JXPW01052892.1	5735-9302	600	3567	34.6	69.3	1.2×10^{-99}	*Totiviridae*	Leptopilina boulardi Toti-like virus	Capsid	Capsid	881-1192	YP_009072447.2	Stratiomyidae	*Hermetia illucens*	Unnamed protein product	CAD7090177.1
np§	72.6	72.6	1289	1	97,208,027-97,209,794	1	GCA_001014895.1	TotiEVE2G1	Forward	JXPW01318876.1	130-1861	295	1731	38.9	88.5	2.6×10^{-51}	*Totiviridae*	Linepithema humile toti-like virus 1	RdRP	RdRP	636-928	AXA52554.1	Stratiomyidae	*Hermetia illucens*	Unnamed protein product	CAD7090177.1
T3	86.2	86.2	1080	6	10,621,536-10,622,617	1	GCA_001014895.1	TotiEVE13G1	Reversed	JXPW01168285.1	326-1578	389	1252	33.2	85.3	9.3×10^{-56}	*Totiviridae*	Leptopilina boulardi Toti-like virus	Capsid	Capsid	1102-1461	YP_009072447.2	Stratiomyidae	*Hermetia illucens*	Unnamed protein product	CAD7092158.1
PR2	100	100	332	2	144,484,848-144,485,179	1	GCA_001014895.1	ParvoEVE1G1	Forward	JXPW01295709.1	732-1063	107	331	45.3	66.4	3.8×10^{-15}	*Parvoviridae*	Haematobia irritans densovirus	VP	Capsid	358-457	QCH41363.1	Uncultured bacterium clone	uncultured bacterium	Hypothetical protein partial	AMP56304.1
DNM	DNM	DNM	DNM	DNM	DNM	2	GCA_009835165.1	ArteriEVE1G2	Forward	VFFH01000119.1	3-3802	1266	3799	97.8	NA	0	*Arteriviridae*	Porcine reproductive and respiratory syndrome virus	PP1a	Non-structural protein	954-2218	AFS30922.1	NA	NA	NA	NA
DNM	DNM	DNM	DNM	DNM	DNM	2	GCA_009835165.1	ArteriEVE2G2	Forward	VFFH01000203.1	1590-2830	199	1240	97.5	NA	1.8×10^{-137}	*Arteriviridae*	Porcine reproductive and respiratory syndrome virus	Major envelope glyoprotein	Major envelope glyoprotein	2-200	AEI72892.1	NA	NA	NA	NA
DNM	DNM	DNM	DNM	DNM	DNM	2	GCA_009835165.1	ArteriEVE3G2	Forward	VFFH01000203.1	4-1575	290	1571	96.6	NA	5.5×10^{-205}	*Arteriviridae*	Porcine reproductive and respiratory syndrome virus	GP3	Envelope protein	1-289	AKZ66193.1	NA	NA	NA	NA
DNM	DNM	DNM	DNM	DNM	DNM	2	GCA_009835165.1	ArteriEVE4G2	Forward	VFFH01001290.1	156-3079	994	2923	93.8	NA	0	*Arteriviridae*	Porcine reproductive and respiratory syndrome virus	Non-structural protein	Non-structural protein	2-995	AFK09091.1	NA	NA	NA	NA
DNM	DNM	DNM	DNM	DNM	DNM	2	GCA_009835165.1	ArteriEVE5G2	Forward	VFFH01002018.1	4-1507	344	1503	99.1	NA	4.3×10^{-245}	*Arteriviridae*	Porcine reproductive and respiratory syndrome virus	RdRP partial	RdRP	1-344	AFX60866.1	NA	NA	NA	NA
DNM	DNM	DNM	DNM	DNM	DNM	2	GCA_009835165.1	ArteriEVE6G2	Forward	VFFH01002425.1	4-3333	1001	3329	99.4	NA	0	*Arteriviridae*	Porcine reproductive and respiratory syndrome virus	RdRP partial	RdRP	460-1460	AFK09092.1	NA	NA	NA	NA
Rh	92.1	92.1	175	2	4,237,406-4,237,585	2	GCA_009835165.1	RhabdoEVE1G2	Forward	VFFH01000694.1	2885224-2885413	49	189	55.1	61.8	6.4×10^{-06}	*Rhabdoviridae*	Entomophthora rhabdovirus A	RdRP	RdRP	546-594	QED42948.1	Plutellidae	*Plutella xylostella*	Uncharacterized protein LOC119694323	XP_037976328.1
PR1	96.3	96.3	231	2	21,909,311-21,909,549	2	GCA_009835165.1	ParvoEVE2G2	Forward	VFFH01002420.1	17403-17642	33	239	66.7	75	1.4×10^{-06}	*Parvoviridae*	Clinch densovirus 1	Hypothetical protein	Capsid	144-187	QNL09579.1	Ixodidae	*Ixodes scapularis*	Uncharacterized protein LOC115327829	XP_029844753.1
np§	49.9	49.9	702	2	144,460,116-144,461,512	2	GCA_009835165.1	PartitiEVE3G2	Forward	VFFH01000694.1	2871386-2872788	284	1402	47.5	30	5.6×10^{-86}	*Partitiviridae*	Atrato Partiti-like virus 2	Capsid	Capsid	101-384	QHA33903.1	Halictidae	*Nomia melanderi*	Uncharacterized protein LOC116434784 isoform X2	XP_031849418.1
PT	94.6	94.6	822	2	144,460,511-144,461,379	2	GCA_009835165.1	PartitiEVE4G2	Forward	VFFH01002716.1	5535-6390	199	855	54.8	31.6	1.7×10^{-59}	*Partitiviridae*	Atrato Partiti-like virus 2	Capsid	Capsid	131-328	QHA33903.1	Halictidae	*Nomia melanderi*	Uncharacterized protein LOC116434784 isoform X2	XP_031849418.1
T1	99.5	99.5	3733	1	97,208,286-97,212,028	2	GCA_009835165.1	TotiEVE3G2	Forward	VFFH01002277.1	524489-528239	622	3750	53	57.1	4.8×10^{-171}	*Totiviridae*	Leptopilina boulardi Toti-like virus	RdRP	RdRP	1-513	YP_009072448.1	Apidae	*Ceratina calcarata*	Uncharacterized protein LOC108628409 partial	XP_017885805.2
np§	74.9	74.9	966	1	97,208,027-97,209,308	2	GCA_009835165.1	TotiEVE10G2	Forward	VFFH01001390.1	32171-33459	336	1288	38.5	85.7	5.7×10^{-88}	*Totiviridae*	Leptopilina boulardi Toti-like virus	Capsid	Capsid	871-1301	YP_009072447.2	Stratiomyidae	*Hermetia illucens*	Unnamed protein product	CAD7090177.1
T2	100	100	3365	5	31,694,664-31,698,028	2	GCA_009835165.1	TotiEVE8G2	Forward	VFFH01001437.1	1443067-1446431	662	3364	30.4	100	8.7×10^{-115}	*Totiviridae*	Leptopilina boulardi Toti-like virus	Capsid	Capsid	870-1192	YP_009072447.2	Stratiomyidae	*Hermetia illucens*	Unnamed protein product	CAD7090177.1
T3	100	100	1211	6	10,621,515-10,622,725	2	GCA_009835165.1	TotiEVE11G2	Reversed	VFFH01001777.1	322470-323680	368	1210	36.7	100	1.4×10^{-66}	*Totiviridae*	Leptopilina boulardi Toti-like virus	Capsid	Capsid	1095-1496	YP_009072447.2	Stratiomyidae	*Hermetia illucens*	Unnamed protein product	CAD7092158.1
DNM	N/A	N/A	N/A	DNM	DNM	2	GCA_009835165.1	ParvoEVE3G2	Forward	VFFH01002716.1	27731-27993	87	262	62.5	69	2.8×10^{-28}	*Parvoviridae*	Haematobia irritans densovirus	NS1	Non-structural protein	140-219	QCH41361.1	Uncultured bacterium clone	uncultured bacterium	Hypothetical protein partial	AMP56273.1
np§	69.1	69.1	493	2	144,484,490-144,485,009	2	GCA_009835165.1	ParvoEVE4G2	Forward	VFFH01002716.1	22618-23330	232	712	33.5	56.9	1.2×10^{-13}	*Parvoviridae*	Haematobia irritans densovirus	VP	Capsid	420-601	QCH41363.1	Uncultured bacterium clone	uncultured bacterium	Hypothetical protein partial	AMP54148.1
PR3	100	100	149	6	3,976,151-3,976,299	3	GCF_905115235.1	ParvoEVE5G3	Forward	LR899014.1	3976151-3976299	48	148	45.8	NA	8.2×10^{-05}	*Parvoviridae*	Lone star tick densovirus 1	NS1	Non-structural protein	187-234	ASU47551.1	NA	NA	NA	NA
PR1	100	100	239	2	21,909,312-21,909,550	3	GCF_905115235.1	ParvoEVE6G3	Forward	LR899010.1	21909312-21909550	79	238	39.2	46.8	2.2×10^{-10}	*Parvoviridae*	Densovirinae sp.	Structural protein partial	Capsid	136-217	ASY04026.1	Ixodidae	*Ixodes scapularis*	Uncharacterized protein LOC115327829	XP_029844753.1
T1	100	100	3743	1	97,208,286-97,212,028	3	GCF_905115235.1	TotiEVE4G3	Forward	LR899009.1	97208286-97212028	1340	3742	35.4	100	1.4×10^{-204}	Unclassified (*Totiviridae* -like)	Dumyat virus	RdRP	RdRP	4-1306	QAY29251.1	Stratiomyidae	*Hermetia illucens*	Unnamed protein product	CAD7078330.1
PT	100	100	1023	2	144,460,311-144,461,333	3	GCF_905115235.1	PartitiEVE5G3	Forward	LR899010.1	144460311-144461333	209	1022	54.1	34.9	8.8×10^{-65}	*Partitiviridae*	Atrato Partiti-like virus 2	Capsid	Capsid	131-339	QHA33903.1	Erebidae	*Arctia plantaginis*	Unnamed protein product	CAB3222417.1
Xi	100	100	1292	5	111,581,535-111,582,826	3	GCF_905115235.1	XinmoEVE1G3	Forward	LR899013.1	111581535-111582826	430	1291	61.6	100	1.9×10^{-192}	*Xinmoviridae*	Lepidopteran anphe-related virus OKIAV50	RdRP	RdRP	12-443	QMP82287.1	Stratiomyidae	*Hermetia illucens*	Unnamed protein product	CAD7091699.1
T2	100	100	3365	5	31,694,664-31,698,028	3	GCF_905115235.1	TotiEVE9G3	Forward	LR899013.1	31694664-31698028	662	3364	30.4	100	8.7×10^{-115}	*Totiviridae*	Leptopilina boulardi Toti-like virus	Capsid	Capsid	870-1192	YP_009072447.2	Stratiomyidae	*Hermetia illucens*	Unnamed protein product	CAD7090177.1
T3	100	100	1199	6	10,621,516-10,622,714	3	GCF_905115235.1	TotiEVE12G3	Forward	LR899014.1	10621516-10622714	365	1198	36.8	100	5.9×10^{-66}	*Totiviridae*	Leptopilina boulardi Toti-like virus	Capsid	Capsid	1096-1496	YP_009072447.2	Stratiomyidae	*Hermetia illucens*	Unnamed protein product	CAD7092158.1
PR2	100	100	332	2	144,485,849-144,485,180	3	GCF_905115235.1	ParvoEVE7G3	Forward	LR899010.1	144484849-144485180	107	331	45.4	66.4	8.1×10^{-10}	*Parvoviridae*	Haematobia irritans densovirus	VP	Capsid	357-457	QCH41363.1	Uncultured bacterium clone	uncultured bacterium	Hypothetical protein partial	AMP56304.1

DMN indicates "did not map"; NA represents "not applicable"; np§ (no position) indicates the orthology was not confirmed and could not be placed on BGA3

Table S3 List of sequences resulting from study and related transcriptome list. (a) contigs of HiTV1 which are longer than 5000 nt in length in cDNA and RNA forms. (b) Short transcripts which contained/mapped to the RdRP 4-like conserved domain detected in BSF transcriptomes and closely related to TotiEVE site T1. (c) Sequenes of short PCR amplified products which targeted different regions of TotiEVE site T1. (d) Sequences of EVE candidates obtained from BGA1, 2 and 3. (e) Regions of BSF genome which flanked the sites on the genome where the sequences of EVE candidates could map. (f) List of transcriptomes where contigs mapped to HiTV1 contig 1.

a

**Name**	**NCBI dataset accession**	**Sequence**	**Genetic sequence form**
HiTV1-contig-1	SRR14339788	[illegible]	cDNA
HiTV1-contig-1-RNA	SRR14339788	[illegible]	RNA
HiTV1-contig-2	SRR10158821	[illegible]	cDNA
HiTV1-contig-2-RNA	SRR10158821	[illegible]	RNA
HiTV1-contig-3	SRR14339795	[illegible]	cDNA

HiTV1-contig-3-RNA	SRR14339795	[illegible]	RNA
HiTV1-contig-4	ERR1801992	[illegible]	cDNA
HiTV1-contig-4-RNA	ERR1801992	[illegible]	RNA
HiTV1-contig-5	SRR8242288	[illegible]	cDNA
HiTV1-contig-5-RNA	SRR8242288	[illegible]	RNA

b

**Name**	**NCBI dataset accession**	**Sequence**	**Genetic sequence form**
TottiEVE-ST1	SRR10158821	CTCCGCCGCAGGAATTCCCCTCGCCATGTAGAAATATAGGTCTTCATCTGATGCACCTCGTACCCGATTTTTTGGTACGTATCTATTGCGGTTTTTACGAATAGTTCAGAGTTCGTGGAAAATATAACATCATCCCCCTGAGCATAGAAATTACCTATGTAAACCTTCTTTCCTATCCTATCCATCGAAATCCTCGCAATCACCCGTAAAGAACACACATTCAAAATGGTATCGAGGACGGTT	cDNA
TottiEVE-ST1	SRR10158821	CUCCGCCGCAGGAAUUCCCCUCGCCAUGUAGAAAUAUAGGUCUUCAUCUGAUGCACCUCGUACCCGAUUUUUUGGUACGUAUCUAUUGCGGUUUUUACGAAUAGUUCAGAGUUCGUGGAAAAUAUAACAUCAUCCCCCUGAGCAUAGAAAUUACCUAUGUAAACCUUCUUUCCUAUCCUAUCCAUCGAAAUCCUCGCAAUCACCCGUAAAGAACACACAUUCAAAAUGGUAUCGAGGACGGUU	RNA
TottiEVE-ST1-(reversed)	SRR10158821	AACCGTCCTCGATACCATTTTGAATGTGTGTTCTTTACGGGTGATTGCGAGGATTTCGATGGATAGGATAGGAAAGAAGGTTTACATAGGTAATTTCTATGCTCAGGGGGATGATGTTATATTTTCCACGAACTCTGAACTATTCGTAAAAACCGCAATAGATACGTACCAAAAAATCGGGTACGAGGTGCATCAGATGAAGACCTATATTTCTACATGGCGAGGGGAATTCCTGCGGCGGAG	cDNA
TottiEVE-ST1-(reversed)	SRR10158821	AACCGUCCUCGAUACCAUUUUGAAUGUGUGUUCUUUACGGGUGAUUGCGAGGAUUUCGAUGGAUAGGAUAGGAAAGAAGGUUUACAUAGGUAAUUUCUAUGCUCAGGGGGAUGAUGUUAUAUUUUCCACGAACUCUGAACUAUUCGUAAAAACCGCAAUAGAUACGUACCAAAAAAUCGGGUACGAGGUGCAUCAGAUGAAGACCUAUAUUUCUACAUGGCGAGGGGAAUUCCUGCGGCGGAG	RNA
TottiEVE-ST2	SRR14339789	CAGAGTTCGTGGAAAATATAACATCATCCCCCTGAGCATAGAAATTACCTATGTAAACCTTCTTTCCTATCCTATCCATCGAAATCCTCGCAATCACCCGAAAAGAACACACATTCAAAATGGTATCGAGGACGGTTGTCCACCGCCATCCCGACGGCAGGCCGTTATTCCACTCCCCCCGACAGTCCCCGCATTCCACTTGCGCCCTCTTTACAAACAATGAATCCCATAGTGCAGCCCACACCCCCTCAAAATCTGTCCCCCTGGTGAGGTTCGAGATATACTCTCCTACAGCTAACATCACAGCGGCTATCCACTTCTTGCTTTGGTGTTGATCAAAC	cDNA
TottiEVE-ST2	SRR14339789	CAGAGUUCGUGGAAAAUAUAACAUCAUCCCCCUGAGCAUAGAAAUUACCUAUGUAAACCUUCUUUCCUAUCCUAUCCAUCGAAAUCCUCGCAAUCACCCGAAAAGAACACACAUUCAAAAUGGUAUCGAGGACGGUUGUCCACCGCCAUCCCGACGGCAGGCCGUUAUUCCACUCCCCCCGACAGUCCCCGCAUUCCACUUGCGCCCUCUUUACAAACAAUGAAUCCCAUAGUGCAGCCCACACCCCCUCAAAAUCUGUCCCCCUGGUGAGGUUCGAGAUAUACUCUCCUACAGCUAACAUCACAGCGGCUAUCCACUUCUUGCUUUGGUGUUGAUCAAAC	RNA

TotiEVE-ST2-(reversed)	SRR14339789	[illegible]	cDNA
TotiEVE-ST2-(reversed)	SRR14339789	[illegible]	RNA
TotiEVE-ST3	SRR14339790	[illegible]	cDNA
TotiEVE-ST3	SRR14339790	[illegible]	RNA
TotiEVE-ST4	SRR14339791	[illegible]	cDNA
TotiEVE-ST4	SRR14339791	[illegible]	RNA
TotiEVE-ST4-(reversed)	SRR14339791	[illegible]	cDNA
TotiEVE-ST4-(reversed)	SRR14339791	[illegible]	RNA
TotiEVE-ST5	SRR14339793	[illegible]	cDNA
TotiEVE-ST5	SRR14339793	[illegible]	RNA
TotiEVE-ST6	SRR14339795	[illegible]	cDNA
TotiEVE-ST6	SRR14339795	[illegible]	RNA
TotiEVE-ST6-(reversed)	SRR14339795	[illegible]	cDNA
TotiEVE-ST6-(reversed)	SRR14339795	[illegible]	RNA

c

**Name**	Larva	**Sequence**	Rearing facility	**Genetic sequence form**
L1-Non-EVE	1	[illegible]	1	DNA
L1-POL-ORF	1	[illegible]	1	DNA
L1-RdRP	1	[illegible]	1	DNA
L2-GAG-ORF	2	[illegible]	2	DNA
L2-Non-EVE	2	[illegible]	2	DNA
L2-POL-ORF	2	[illegible]	2	DNA
L2-RdRP	2	[illegible]	2	DNA
L3-GAG-ORF	3	[illegible]	3	DNA
L3-Non-EVE	3	[illegible]	3	DNA
L3-POL-ORF	3	[illegible]	3	DNA
L3-RdRP	3	[illegible]	3	DNA

d

**Name**	**Contig NCBI accession**	**Sequence**	**Coordinates on contig**	**Genetic sequence form**
NA	VFFH01000119.1	[illegible]	3 - 3802	DNA
NA	VFFH01000203.1	[illegible]	1590 - 2830	DNA
NA	VFFH01000203.1	[illegible]	4 - 1575	DNA
NA	VFFH01001290.1	[illegible]	156 - 3079	DNA
NA	VFFH01002018.1	[illegible]	4 - 1507	DNA

NA	VFFH01002425.1	[illegible]	4 - 3333	DNA
PartitiEVE1G1	JXPW01014295.1	[illegible]	343 - 1512	DNA
PartitiEVE2G1	JXPW01121853.1	[illegible]	492 - 1707	DNA
PartitiEVE3G2	VFFH01000694.1	[illegible]	2871386 - 2872788	DNA
PartitiEVE4G2	VFFH01002716.1	[illegible]	5535 - 6390	DNA
PartitiEVE5G3	LR899010.1	[illegible]	144460311 - 144461333	DNA
ParvoEVE1G1	JXPW01295709.1	[illegible]	732 - 1063	DNA
ParvoEVE2G2	VFFH01002420.1	[illegible]	17403 - 17642	DNA
ParvoEVE3G2	VFFH01002716.1	[illegible]	27731 - 27993	DNA
ParvoEVE4G2	VFFH01002716.1	[illegible]	22618 - 23330	DNA
ParvoEVE5G3	LR899014.1	[illegible]	3976151 - 3976299	DNA
ParvoEVE6G3	LR899010.1	[illegible]	21909312 - 21909550	DNA
ParvoEVE7G3	LR899010.1	[illegible]	144484849 - 144485180	DNA
RhabdoEVE1G2	VFFH01000694.1	[illegible]	2885224 - 2885413	DNA
TotiEVE1G1	JXPW01237450.1	[illegible]	1885 - 3346	DNA
TotiEVE2G1	JXPW01318876.1	[illegible]	130 - 1861	DNA
TotiEVE3G2	VFFH01002277.1	[illegible]	524489 - 528239	DNA
TotiEVE4G3	LR899009.1	[illegible]	97208286 - 97212028	DNA
TotiEVE5G1	JXPW01175605.1	[illegible]	2029 - 3362	DNA
TotiEVE5G1-(reversed)	JXPW01175605.1	[illegible]	3362 - 2029	DNA
TotiEVE6G1	JXPW01052892.1	[illegible]	5735 - 9302	DNA
TotiEVE6G1-(reversed)	JXPW01052892.1	[illegible]	9302- 5735	DNA
TotiEVE7G1	JXPW01318472.1	[illegible]	69 - 1591	DNA

TotiEVE8G2	VFFH01001437.1	[illegible]	1443067 - 1446431	DNA
TotiEVE9G3	LR899013.1	[illegible]	31694664 - 31698028	DNA
TotiEVE10G2	VFFH01001390.1	[illegible]	32171 - 33459	DNA
TotiEVE11G2	VFFH01001777.1	[illegible]	322470 - 323680	DNA
TotiEVE11G2-(reversed)	VFFH01001777.1	[illegible]	323680 - 322470	DNA
TotiEVE12G3	LR899014.1	[illegible]	10621516 - 10622714	DNA
TotiEVE13G1	JXPW01168285.1	[illegible]	326 - 1578	DNA
TotiEVE13G1-(reversed)	JXPW01168285.1	[illegible]	1578 - 326	DNA
XinmoEVE1G3	LR899013.1	[illegible]	111581535 - 111582826	DNA

e

Site	NCBI dataset accession	Sequence	Coordinates on chomosomal sequence	Genetic sequence form
T1 - chromosome 1	NC-051849.1	[illegible]	97,200,000 - 97,220,000	DNA

Rh - chromosome 2	NC-051850.1	[illegible]	4,227,641 - 4,247,641	DNA
PR1 - chromosome 2	NC-051850.1	[illegible]	21,899,609 - 21,919,609	DNA

PT - chromosome 2	NC-051850.1	[illegible]	144,451,311 - 144,471,311	DNA
PR2 - chromosome 2	NC-051850.1	[illegible]	144,475,520 - 144,495,520	DNA

T2 - chromosome 5	NC-051853.1	[illegible]	31,686,654 - 31,706,654	DNA
Xi - chromosome 5	NC-051853.1	[illegible]	111,572,710 - 11,592,710	DNA

PR3 - chromosome 6	NC-051854.1	[illegible]	3,965,840 - 3,985,840	DNA
T3 - chromosome 6	NC-051854.1	[illegible]	10,612,027 - 10,632,027	DNA

f

List of transcriptomes where contigs mapped to HiTV1 contig 1:

SRR14339796	SRR8242285
SRR14339795	SRR8242284
SRR14339794	SRR8242283
SRR14339793	SRR8242282
SRR14339791	SRR8242281
SRR14339790	SRR8242280
SRR14339789	SRR8242279
SRR14339788	SRR8242277
SRR14339787	SRR6656088
SRR14339786	SRR6656087
SRR14339785	ERR1801998
SRR14339784	ERR1801997
SRR14339783	ERR1801996
SRR14339782	ERR1801995
SRR10233312	ERR1801994
SRR10158821	ERR1801993
SRR8242297	ERR1801992
SRR8242293	ERR1801991
SRR8242292	ERR1801990
SRR8242291	ERR1801989
SRR8242289	ERR1801988
SRR8242288	ERR1801987
SRR8242287	ERR1801986
SRR8242286	ERR1801985
